# The Biocomplex Assembled from Antigen Peptide and Toll-like Receptor Agonist Improved the Immunity against Pancreatic Adenocarcinoma *In Vivo*

**DOI:** 10.1155/2022/2965496

**Published:** 2022-08-25

**Authors:** Wenming Feng, Hongbin Yu, Tao Xue, Chunyong Wu, Fan Ren, Ge Cui

**Affiliations:** ^1^Department of General Surgery, The First Affiliated Hospital, Huzhou University, Huzhou, China; ^2^Department of Medical Therapeutics, The First Affiliated Hospital, Huzhou University, Huzhou, China; ^3^Research Center, The First Affiliated Hospital, Huzhou University, Huzhou, China; ^4^Clinical Laboratory, The First Affiliated Hospital, Huzhou University, Huzhou, China; ^5^Department of Pathology, The First Affiliated Hospital, Huzhou University, Huzhou, China

## Abstract

**Purpose:**

One of the biggest challenges in cancer immunotherapy is generating robust cancer-specific immunity. This work describes using a biocomplex assembled from a toll-like receptor agonist CpG oligodeoxynucleotide 1826 (CpG) and a pancreatic cancer antigen peptide mesothelin for tuning pancreatic tumor immunity.

**Methods:**

This biocomplex was assembled via electrostatic interactions and characterized in size, morphology, zeta potential, and cargo loading. The effect of biocomplex on cell viability and activation of DCs and macrophages were measured by flow cytometry. The production of cytokines (GM-CSF, TNF, and IL-6) was evaluated by using ELISA kits. The effect of biocomplex on tumor cell proliferation was also evaluated by *in vivo* tumor model.

**Result:**

We can modulate the surface charge of the biocomplex by simply varying the ratios of the two components. In cell models, this biocomplex did not impact cell viability in the antigen-presenting cell (i.e., dendritic cell and macrophage)-directed immunity. Moreover, this biocomplex regulated the secretion of tumor-related cytokines (i.e., GM-CSF, TNF, and IL-6) and promoted the activation of immune cell surface markers (i.e., CD80+, CD86+, and CD40+). In the mouse model, the biocomplex inhibited the tumor burden effectively and promoted the production of effector cytokines.

**Conclusion:**

The present studies showed that the biocomplex with antigen peptide and toll-like receptor agonist was able to potentiate the antitumor immunity *in vivo*. This study will help understanding of immunity in pancreatic cancer and developing new immune therapeutic strategies for pancreatic adenocarcinoma.

## 1. Background

Pancreatic cancer remains one of the most aggressive and lethal tumors [1, 2]. Current clinical treatment relies on using multimodal therapies, including surgical resection, radiotherapy, and cytotoxic chemotherapy, or a combination of these therapies. However, the 5-year survival rate of patients with pancreatic cancer is still less than 5% [3]. Currently, immunotherapy is one of the most promising modalities for treating different types of cancers, including pancreatic adenocarcinoma. However, multiple challenges hinder the development of effective immunotherapeutic strategies [4, 5]. In particular, researchers are facing the difficulty of conquering the robust immune-suppressive environment generated by the tumor tissue, lacking the capacity to promote a vigorous and tumor-specific immunity against cancer [6, 7]. Thus, it is necessary to explore novel strategies that can boost healthy and specific immunity against cancers.

Biomaterial-based techniques have been broadly explored to enhance the efficacy of therapeutic cargo delivery for treating different diseases [8–11]. Compared to conventional technologies, biomaterial-based strategies possess dramatic advantages, including controlled release of cargos, targeting, and co-delivery of therapeutic agents. For immune modulation purposes, biomaterials allow incorporating immune-stimulatory or suppressive cargos, enabling a prolonged release of these cargos that can continuously stimulate immune functions [9, 10]. Additionally, biomaterials also allow targeting specific immune cells or organs that can produce robust and specific cancer immunity with minimum side effects as compared to traditional therapies [12, 13]. Through controlling parameters such as size and shape, immune cargos can be effectively guided to immune organs such as the lymph nodes and spleen, thus promoting potent immunity that can be harvested to combat different immune-associated diseases [14–16]. Broadly explored biomaterial-based techniques include using polymeric materials such as poly(lactic-co-glycolic acid), polycaprolactone, or lipid to incorporate immune cargos that allow sustained release of the agents to achieve a targeting effect [12]. Different strategies also involve using thin-film structures in the formation of multilayered capsules that incorporate immunological signals within the film or the hollow capsules [17–19]. Additionally, another simple biomaterial-based method is to directly condense immune cargos such as peptides and adjuvant materials into biocomplex [20–22]. Compared to other technologies, the biocomplex technique is simple, direct, and avoids contacting the immune signals with harmful organic solvents, thus preserving the immunogenicity of the cargos [20–22]. Thus, this technology was employed in our work for modulating the cancer-specific immunity against pancreatic cancer.

Toll-like receptor (TLR) agonists such as CpG (CpG oligodeoxynucleotide 1826), a TLR-9 agonist, are DNAs or RNAs that can bind and activate toll-like receptors in the immune cells. In the immune system, the toll-like receptors participate in detecting pathogens such as viral RNA and bacteria peptide that is not commonly existed in mammals [23–25]. Once the immune cells encounter the danger signals, for example, toll-like receptors in the innate and adaptive immune system are activated. Based on these immunological reactions, toll-like receptors are actively explored in multiple clinical and preclinical trials for the treatment of various cancers [26–28]. While these trials are promising, striking discoveries in recent years found that a combination of toll-like receptor agonists with the tumor antigens can promote potent antigen-specific immunity that can be explored to destroy the immune-suppressive cancer environment [29–31].

In this work, a biocomplex was generated by combining a toll-like receptor with a tumor-associated antigen peptide. The antigen peptide was modified to facilitate an active interaction between the two cargos. To investigate its antitumor potency and mechanisms, we used a biocomplex assembled from a toll-like receptor agonist CpG oligodeoxynucleotide 1826 (CpG) and a pancreatic cancer antigen peptide mesothelin for tuning pancreatic tumor immunity in *in vivo* assays. The study indicated that the biocomplex generated from a toll-like receptor agonist and a tumor antigen peptide could be employed as a simple, direct, and robust strategy for modulating the tumor immunity in the pancreatic cancer model.

## 2. Materials and Methods

### 2.1. Materials

1× phosphate-buffered saline (PBS) and Chitosan (MW = 20000) were purchased from Sigma. DAPI (4-,6-diamidino-2-phenylindole) was purchased from Invitrogen. The positive isolation beads for isolating dendritic cells were purchased from Miltenyi Biotec. Fluorescently labeled antibodies for CD80 (PE), CD86 (PE-Cy7), and CD40 (APC) were purchased from Biolegend. RPMI cell culture medium was obtained from VWR. Mesothelin peptide was synthesized and purchased from Tiangong Biotech. Incorporation.

### 2.2. Cells and Animals

All animal experiments were approved by the Animal Research Committee Board of Huzhou University, and animal experiments were performed by following the Institutional Animal Care and Use Committee (IACUC). Mice were sacrificed by exposing to CO_2_ with gradually increased concentration. The cervical dislocation was employed to ensure a successful sacrifice. Dulbecco's modified eagle's or RPMI medium plus 10% fetal calf serum was used for cell culture. The method for generating a solid tumor in mice was established. Briefly, 1 × 10^7^ pancreatic cancer cells (PANC-1) were implanted subcutaneously into the flanks of mice (female mice, 6–7 weeks old, ∼20 g for each mouse). A tumor was established to sizes ranging from 100 mm^3^ to 150 mm^3^ in 10 to 15 days. Blood samples were collected, and CD8+ T cells were analyzed by a flow cytometer. IL-6, IFN*ɤ*, and IL-10 were measured by the ELISA test.

### 2.3. Biocomplex Synthesis

The biocomplex assembled from CpG and mesothelin peptide was produced by mixing the two cargos at different N : P ratios (i.e., 10 : 1, 5 : 1, 3 : 1, 1 : 1, 1 : 3, 1 : 5, and 1 : 10), where N : P ratios mean the number of amine groups on the cationic R amino acid-modified peptide and the number of phosphate (P) groups on the negatively charged CpG. CpG at a concentration of 5 *μ*g/mL maintained at a fixed volume was used to prepare the complex by adding a varied amount of peptide into the solution. By doing this, we can control the amount of CpG in different types of biocomplex. The biocomplex was stored in 1× PBS buffer at pH 7.0. For *in vitro* and *in vivo* studies, the biocomplex was freshly prepared and used immediately before the study.

### 2.4. Biocomplex Characterization

The biocomplex was characterized by their hydrodynamic diameter and zeta potential. The size and zeta potential of the biocomplex were measured by using Malvern Zetasizer Nano ZS90 (Westborough, MA, USA). The test was performed in PBS immediately after biocomplex preparation.

### 2.5. *In vitro* Test

For the cell viability test, the mouse splenic dendritic cells were collected. Briefly, the spleen was minced to a size smaller than 1 mm^2^, followed by treatment with dissociation medium (Miltenyi Biotec) and collected by positive magnetic collection (CD11c antibody coated with magnetic beads). Then, CD11c-positive DCs were treated with different biocomplex samples. DCs were treated with soluble CpG (5 ug/ml), peptide (10 *μ*g/ml), or a mixture of CpG (5 ug/ml), and peptide (10 *μ*g/ml). PBS was used as a control (CTRL group). The treatment lasted for 24 hours, followed by staining with AO/PI and assessed with flow cytometry to test their viability. Commercial macrophages RAW264.7 were used as another cell line for viability and activation tests. The activation of DCs and macrophages was examined by staining the cells with fluorescently labeled antibodies (CD80^+^, CD86^+^, and CD40^+^) (Invivogen) and assessed via flow cytometry. The production of cytokines from macrophages or DCs was evaluated using ELISA kits by following the manufacturer's instructions. We followed the methods of Miao et al. [32].

### 2.6. ELISA Test

To test the level of cytokines in the mouse serum, C57/BL6 mice were immunized with different samples (CpG + Meso (toll-like receptor agonist (CpG)+mesothelin), biocomplex, CpG, Meso(mesothelin), or soluble mixture) on day 0, and peripheral blood was collected from mice on day 3. The blood was centrifuged at 1800 G for 15 min to collect the serum. The serum was stored for the ELISA test by following the manufacturer's instructions.

### 2.7. Tumor Study

C57/BL6 mice (female, 4–8 week old, *n* = 40) were immunized with different samples (intratumoral injection) (i.e., biocomplex, CpG, peptide, or soluble mixture; each group has 5 C57/BL6 mice) on day 0, followed with a boost on day 15. The mice were implanted with 3 × 10^5^ pancreatic cancer cells on the flank of mice on day 16. Tumor size was measured every day and volume determined by using Width^2^ × Length. Mice were sacrificed when the tumor size reached 150 mm^3^.

## 3. Results

### 3.1. Characterization of the Biocomplex Assembled from CpG and Mesothelin Peptide

**Figure 1:**
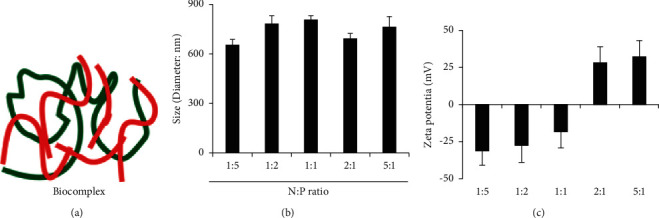
Characterization of the biocomplex assembled from CpG and mesothelin peptide. (a) A schematic picture showing the structure of the biocomplex. (b) The size distribution of the biocomplex at different *N* : *P* ratio. (c) Zeta potential measurement of the biocomplex with different *N* : *P* ratios.

The first task in our study was to generate the biocomplex by integrating the two immune signals (CpG and mesothelin peptide) via electrostatic interactions (Figure 1(a)). The two immune signals were condensed into a biocomplex due to its electrostatic interactions. We evaluated the impact of different N : P ratio ranging from 1 : 5 to 5 : 1 on the size and zeta potential of the biocomplex (Figures 1(b) and 1(c)). To ensure a consistent amount of CpG in our study, we used a fixed amount of negatively charged CpG in the test and varied the amount of positively charged R6-modified peptide. The N : P ratio was employed as a parameter to help analyze the relative ratio of the peptide (N) to the negatively charged phosphate group in the backbone of CPG. A study with dynamic light scattering indicated the production of biocomplex (Figure 1(b)). The size of the biocomplex with N : P ratio of 1 : 5, 1 : 2, 1 : 1, 2 : 1, and 5 : 1 was 650.9 ± 35.2, 780.5 ± 50.8, 800.0 ± 30.1, 696.0 ± 34.5, and 760.0 ± 66.9 nm, respectively. There was no dramatic difference between the size of the biocomplex, but the size distribution fits the diameter ranges for *in vivo* circulation. For the interaction between cells, especially immune cells such as macrophage and dendritic cells, the size range allows efficient internalization, cellular processing, and presentation of the peptide and adjuvant materials.

Zeta potential study indicated that the relative N : P ratio impacts the surface charges significantly. For a lower N : P ratio, such as 1 : 5, the biocomplex showed a negatively charged surface. As a contrast, a higher N : P ratio, such as 5 : 1, the biocomplex showed a positively charged surface. Specifically, the biocomplex with N : P ratio of 1 : 5, 1 : 2, 1 : 1, 2 : 1, and 5 : 1 had surface charge of −31.2 ± 10.5, −27 ± 12.1, −18.5 ± 9.1, 28.9 ± 10.5, and 32.2 ± 10.3, respectively (Figure 1(c)). This result thus illustrates that we can modulate the surface charge of the biocomplex by simply varying the ratios of the two components. With the amount of CpG fixed, this technique allowed us to study the impact of surface charges and peptide loading on the therapeutic effect of the biocomplex. Also, the negatively charged surfaces may allow the biocomplex to have a prolonged circulation time *in vivo*, thus enabling an enhanced therapeutic effect by avoiding the fast clearance of the particles within the body by organs such as the kidney and lung.

### 3.2. The Effect of the Biocomplex on Principal Immune Cells

**Figure 2:**
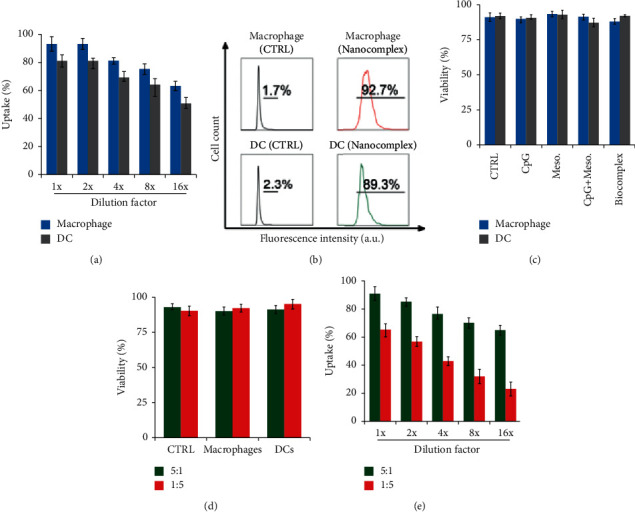
*In vitro* characterization of the biocomplex to principal immune cells, that is, macrophage and DC. (a) Uptake of the biocomplex by macrophage and DC as characterized by flow cytometry. The biocomplex was diluted by different ratios to test the internalization rate at different concentrations. (b) Fluorescence intensity characterizing the internalization of 1× biocomplex. (c) Impact of biocomplex, as well as its component, to the viability of the immune cells. (d) The effect of different biocomplex with different *N* : *P* ratios (1 : 5 and 5 : 1) to macrophage and DC viability. (e) The uptake of the biocomplex with different *N* : *P* ratios by macrophages and DCs. CTRL : Control (treated with PBS).

The biocomplex was then utilized to interact with immune cells, including DCs and macrophages. The modified biocomplex was designed for intradermal (i.d.) injection, where dendritic cells and macrophages would be two of the primary cells to take up the biocomplex. Thus, these two cell types were employed for the uptake study. Fluorescently labeled peptide and CpG at an N : P ratio of 1 : 1 was employed to compose the biocomplex to facilitate flow cytometric analysis. The uptake study illustrated that both macrophages and DCs were able to internalize the biocomplex in a dose-dependent manner. By increasing the dilution factor of the biocomplex ranging from 2× to 16×, the uptake of the biocomplex is reduced (Figure 2(a)). The uptake was confirmed by flow cytometric analysis. The fluorescence intensity of the control group is 1.7% (macrophages) and 2.3% (DCs). In addition, both macrophages and DCs showed significant uptake of immune signals, 92.7% and 89.3% for macrophages and dendritic cells, respectively, indicating a majority of cells have internalized the biocomplex (Figure 2(b)). A high internalization efficiency by the cells was vital for delivering the immunological cargos to the antigen-presenting cells. These data demonstrated the feasibility of using the biocomplex as a delivery vehicle for immune cargo transfer. A viability assay was then used to investigate the impact of the biocomplex on the viability of DCs and macrophages. Compared to cells with no treatment, the biocomplex did not cause any toxicity to the dendritic cells nor to the macrophages. Meanwhile, the viability study also illustrated neither CpG nor the peptide caused any toxicity to the cells, indicating the biological safety of the biocomplex (Figure 2(c)).

Since surface property, especially the surface charges, is a critical factor that impacts the interactions between cells and micro/nanomaterials, we analyzed the impact of surface charges on cellular uptake and viability of cells using the biocomplex with *N* : *P* ratio of 1 : 5 and 5 : 1. We found that the biocomplex at *N* : *P*=1 : 5 or *N* : *P*=5 : 1 had no significant toxicity on macrophages and DCs (Figure 2(d)). These data demonstrated that the surface charges are not a substantial concern for the cellular toxicity issue. Compared to the negatively charged biocomplex that had an *N* : *P*=1 : 5, the positively charged biocomplex with *N* : *P*=5 : 1 had a more efficient internalization rate when added with the same amount of biocomplex, indicating that the positively charged biocomplex can be internalized by macrophages more efficiently (Figure 2(e)). This result suggested that the cells prefer positively charged surfaces more than negatively charged surfaces.

### 3.3. The Effect of Biocomplex on the Activation of DC Surface Markers

**Figure 3:**
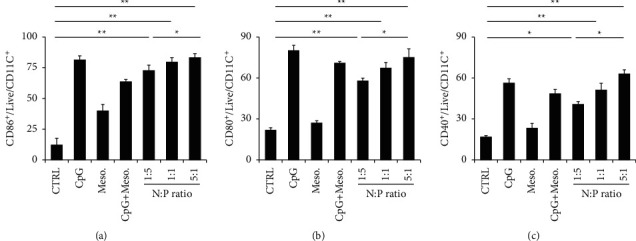
Assessing the impact of biocomplex on the activation of DC surface markers (a) CD86^+^, (b) CD80^+^, and (c) CD40^+^. Biocomplex with different *N* : *P* ratios were employed for the study (*N* : *P*=1 : 5, 1 : 1, and 5 : 1). CTRL : Control (treated with PBS), ^*∗*^*P* < 0.05, ^*∗∗*^*P* < 0.01.

**Figure 4:**
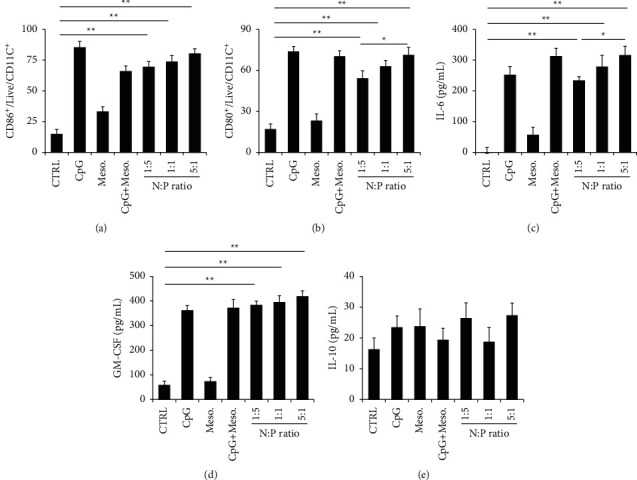
The impact of the biocomplex on macrophage immunity and the influence of the biocomplex on the activation of (a) CD86+ and (b) CD86+ markers. Assessing the secretion of (c) IL-6, (d) GM-CSF, and (e) IL-10 levels in the macrophage culture supernatant. The biocomplex with different *N* : *P* ratios was employed for the study (*N* : *P*=1 : 5, 1 : 1, and 5 : 1). CTRL : Control (treated with PBS), ^*∗*^*P* < 0.05, ^*∗∗*^*P* < 0.01.

Antigen-presenting cells such as DCs and macrophages play vital roles in modulating innate and adaptive immunity. Through internalizing immune-stimulatory signals, including antigens and adjuvants, these cells will be activated and then move to the lymph nodes to promote the activation of naïve B and T cells. This process thus makes the activation of antigen-presenting cells an important topic in studying vaccine efficacy. To investigate the impact of the biocomplex on DCs, we interacted the biocomplex with DCs, focusing on the assessment of DC surface markers (i.e., CD86^+^, CD80^+^, and CD40^+^). Briefly, primary DCs isolated from the mouse spleen were used to interact with the biocomplex and analyzed by flow cytometry. PBS was used as control. Flow cytometric assessments illustrated that the biocomplex promoted CD86+ activation on DC surfaces. 72.5 ± 4.7%, 79.5 ± 3.9%, and 83 ± 3.5% of CD86+ markers were activated in DCs treated by the biocomplex with the *N* : *P* ratio of 1 : 5, 1 : 1, and 5 : 1, respectively (Figure 3(a)). Positively charged biocomplex (i.e., *N* : *P*=5 : 1) activated a higher ratio of CD86+ markers on DCs than the biocomplex with *N* : *P*=1 : 5 or *N* : *P*=1 : 1 (^*∗*^*P* < 0.05 for *N* : *P*=1 : 5 vs. *N* : *P*=5 : 1) The untreated control group had a low activation ratio (i.e., 12.15 ± 5.1%) (Figure 3(a)). Similarly, the peptide led to a low CD86+ activation due to the low immunogenicity of the material. CpG, as a toll-like receptor agonist, caused a high activation ratio in CD86+ surface markers in both the CpG and CpG + Meso. groups (Figure 4(a)). Similar results were observed in the activation of CD80+ and CD40+ cells. The biocomplex with cationic surface property (i.e., *N* : *P*=5 : 1) activated a higher ratio of cells than those with negative ionic surface (*N* : *P*=1 : 5 or *N* : *P*=5 : 1) (^*∗*^*P* < 0.05 for *N* : *P*=1 : 5 vs. *N* : *P*=5 : 1) (Figures 3(b) and 3(c)). This might be caused by the surface property of the biocomplex, where the negatively charged surfaces have a lower internalization rate compared to that of positive charges. The results indicated that surface property plays an important role in regulating the interactions between micro/nanomaterials and DCs.

### 3.4. The Effect of Biocomplex on Macrophage Immunity and the Activation of CD86+ and CD80+ Cells

We further evaluated the impact of the biocomplex on macrophage due to its vital roles in combating tumors. The biocomplex with different *N* : *P* ratios, as well as control samples, was used to interact with macrophages. For macrophage activation, the biocomplex with *N* : *P* ratio of 1 : 5, 1 : 1, and 5 : 1 activated macrophage surface markers (i.e., CD86+ and CD80+). Compared to the biocomplex with negative charges (i.e., *N* : *P*=1 : 1 or 1 : 5), the positively charged biocomplex activated the markers more effectively (Figures 4(a) and 4(b)). A statistical difference was observed in the activation of CD80+ markers between the biocomplex with *N* : *P*=1 : 5 and that with *N* : *P*=5 : 1, indicating the impact of surface charges in the interactions between macrophages and the biocomplexes (Figures 4(a) and 4(b)). Compared to all the biocomplex groups with the CTRL group, there was a statistical importance, showing the effectiveness of the biocomplex in regulating macrophage immunity (*P* < 0.01, Figures 4(a) and 4(b)). Beyond the activation study, the supernatant of the macrophage culture medium was collected for the cytokine study. The level of IL-6, G-CSF, and IL-10 levels was measured (Figures 4(c)–4(e)). All three types of biocomplexes caused increased production of IL-6 and G-CSF cytokines compared to the CTRL group (Figures 4(c) and 4(d)). This result indicated that the use of CpG in the biocomplex caused an inflammatory response, while the G-CSF cytokine would be involved in the antitumor functions. Studying the impact of *N* : *P* ratios, we found that a cationic biocomplex with *N* : *P*=1 : 5 led to a higher level of IL-6 and G-CSF as compared to the anionic biocomplex (*N* : *P*=1 : 1 and *N* : *P*=1 : 5). These data again illustrated the importance of surface property in regulating immunity. The study revealed no significant difference among all the groups in the production of the regulatory cytokines (Figure 4(e)).

### 3.5. *In vivo* Analysis of the Effect of the Biocomplex on Tumor Progression

**Figure 5:**
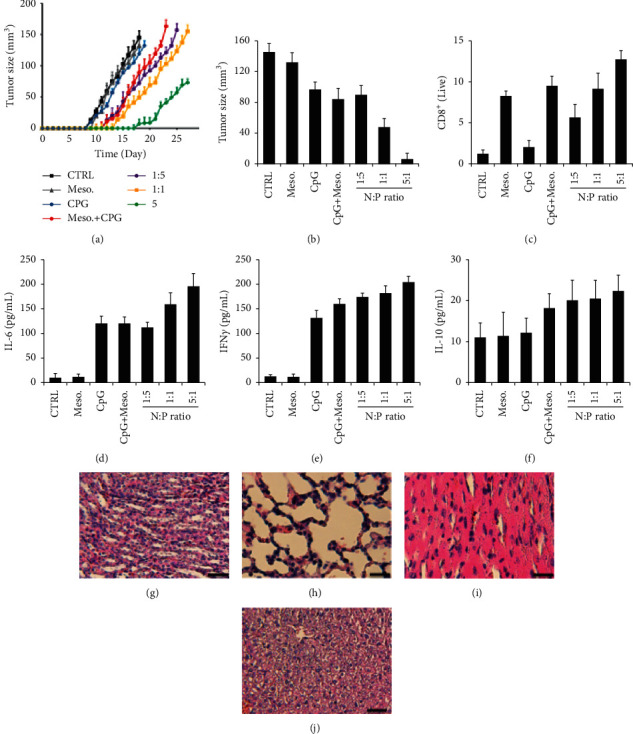
*In vivo* analysis of the impact of the biocomplex on tumor progression in mice. (a) Tumor size in mice over time with different treatments. (b) Tumor size on day 18 in different groups of mice. (c) The level of CD8+ T cells in mice on day 18. The biocomplex with different N : P ratios were employed for the study (*N* : *P*=1 : 5, 1 : 1 and 5 : 1). Assessing the levels of (d) IL-6, (e) IFN*ɤ*, and (f) IL-10. Histological staining showing the impact of the biocomplex on major organs including the (g) kidney, (h) lung, (i) heart, and (j) liver. CTRL : Control (treated with PBS), Scale bar = 50 *μ*m ^*∗*^*P* < 0.05, ^*∗∗*^*P* < 0.01.

For the *in vivo* tumor model study, we pretreated the mice with the vaccine components and then implanted the mice with solid tumors to observe how the tumor evolves. Tumor size measurement indicated a different tumor size over time (Figure 5(a)). The vaccine groups (i.e., *N* : *P*=1 : 5, 1 : 1, and 5 : 1) had a slower tumor growth as compared to the control group (CTRL group) (Figure 5(a)). *N* : *P* ratio seems to affect the tumor progression rate as well, where the vaccine with *N* : *P*=5 : 1 had the slowest tumor progression rate as compared to the vaccines with *N* : *P*=1 : 5 and *N* : *P*=1 : 1 (Figure 5(a)), which is probably caused by different loading of CpG within the vaccines. On day 18, which was the date that all the mice in the CTRL group were sacrificed, we recorded the average tumor size in each group (Figure 5(b)). Tumor size on day 18 indicated a similar tumor progression rate as compared to Figure 5(a). Briefly, the biocomplex groups have a smaller tumor size compared to the control groups, and the *N* : *P*=5 : 1 vaccine group had the smallest tumor size among all the groups (Figure 5(b)). We also collected the peripheral blood of mice on day 18 to measure the level of CD8+ T cells in all the groups (Figure 5(c)). The biocomplex (i.e., *N* : *P*=1 : 5, 1 : 1, and 5 : 1) groups had a higher level of CD8+ T cells. These data partially explained the tumor progression data in Figures 5(a) and 5(b), where a higher level of CD8+ T cells may be responsible for inhibiting tumor progression (Figure 5(c)). In addition to the above studies, we also measured the level of cytokines in mice from different groups on day 18. For the inflammatory cytokines like IL-6, the use of CpG in the treatment promote this cytokine level in the blood of mice (Figure 5(d)). Meanwhile, using CpG also caused an increase in the level of the antitumor cytokines such as IFN*γ*, indicating that CpG plays a vital role in the antitumor functions (Figure 5(e)). For the regulatory cytokines such as IL-10, we did not observe any statistical differences among the groups (Figure 5(f)). Thus, the levels of cytokines in the mouse serum can partially explain the antitumor functions of the cargos, especially for the biocomplex. Using histological staining, we assessed the impact of the biocomplex on major organs of mice, including the kidney, lung, heart, and liver (Figures 5(g)–5(k)). We did not observe any obvious changes to these organs, indicating the biosafety of this biocomplex.

## 4. Discussion

Immune suppression is one of the significant issues associated with immunotherapy of cancers, including pancreatic cancer. Cancer progression is usually associated with impaired immune function, specifically, suppressed effector T-cell function and enhanced regulatory T-cell function [6]. The search for adequate immune cargos to combat these suppressions has become one of the key goals in the treatment of cancers. Toll-like receptor agonists—a group of “danger” signals (i.e., pathogens) that exists widely in viruses and bacteria but not in humans–have been employed in different ways for cancer treatment [33–35]. For example, CpG was conjugated to biocomplex to activate macrophages for the immune therapy of disease; the use of CpG enhanced the secretion of cytokines associated with anticancer procession [36]. In another study, polyIC—a TLR3 agonist—was assembled with a peptide onto the biocomplex to expand antigen-specific T cells *in vivo* [37]. There were also examples of using TLR adjuvants for clinical trials of cancer treatment [33, 34]. As for glioma—one of the most challenging cancers in the world—immune therapies have also drawn extensive attention in recent years. For example, the delivery of genes that encode cytokines that can modulate the immune-suppressive tumor microenvironment has shown promising results by promoting DC activation and effector T-cell proliferation [38]. In a clinical trial, CpG was used in a phase 1 trial to treat patients with recurrent glioblastoma; preliminary evidence of this study found that two in six patients had a median survival period of 7.2 months [39]. Studies also showed that CpG could promote inflammatory cytokine secretion while promoting the level of effector CD8+ T cell, thus showing great potential in combating cancer in several clinical trials [27, 40]. Therefore, we used CpG with a tumor antigen to form biocomplex via electrostatic interactions. Such an assembly allows integrating the two immune signals into one nanoparticle, thus ensuring the two therapeutic cargos will be delivered to the same immune cells and organs compared to injecting a mixture of soluble cargos. Also, using the biocomplex will protect the cargos from being degraded in *in vivo* environment. Previous studies found that the co-delivery of different immune cargos (i.e., different toll-like receptor agonists or antigen plus adjuvant) could generate potent synergistic effects much higher than from using each component alone [37]. This was consistent with what was demonstrated in our study. The antigen alone did not activate DC surface markers (i.e., CD80+ and CD86+) due to its low immunity.

To investigate the *in vivo* functionality of the biocomplex, we treated mice with different samples. The biocomplex was able to prohibit tumor progression as compared to using free cargos. This was consistent with the functional cytokine level (i.e., IFN*γ*), which was responsible for killing the cancer cells. This is probably either because the CpG in particulate form has an improved adjuvant effect, or because of the synergistic effect of co-delivering CpG and the tumor antigen together. We also noticed that antigen alone yielded a low level of IL-6 and IFN*γ*, indicating the peptide was not involved in promoting the production of inflammatory and effector cytokines. These data suggest that the antigen involved was not significantly engaged in the effector but participated in an inflammatory immune response during the vaccination. While the *in vivo* tumor study illustrated a synergistic effect of the complex against pancreatic cancer, these DC activations and *in vivo* and *in vitro* assays partially explained the mechanism underlying this synergistic performance. It is well known that DCs have the capacity of antigen cross-presentation, and many efforts have been made to improve antigen presentation potency against tumor cells through inducing antigen-specific cytotoxic T lymphocyte (CTL) responses. In addition, the combination of DC vaccines with additional therapy (such as chemotherapy and monoclonal antibodies) could confer efficient cancer therapeutics [41].

Previous studies have shown that macrophages can promote cancer initiation and malignant progression by increasing tumor cell migration or suppressing antitumor immunity [42]. For a better understanding of using antigen and CpG for cancer treatment, further study requires the investigation of the role of the B cell—a major immune cell for antibody immunity in these processes. Besides, studies on the activation pathway of immune cells, as well as the infiltration of immune cells into tumor tissues, will also help us understand the mechanism in this study.

In summary, this study investigated the use of a biocomplex composed of CpG and a tumor antigen for cancer treatment. Both *in vitro* and *in vivo* studies showed that the biocomplex was able to potentiate the antitumor immunity. Further studies, such as the infiltration of immune cells to the tumor as well as B-cell immunity, will be performed to better understand the mechanism of using the biocomplex.

## Data Availability

The raw data used to support the findings are available from the corresponding author upon request.
